# Progressive Palaeontology 2024 conference report

**DOI:** 10.1242/bio.061822

**Published:** 2024-12-06

**Authors:** Hady George, Thomas Farrell, James R. G. Rawson, Isaura Aguilar-Pedrayes, Benton Walters, Kirsten E. Flett

**Affiliations:** Bristol Palaeobiology Research Group, School of Earth Sciences, University of Bristol, Life Sciences Building, 24 Tyndall Avenue, Bristol BS8 1TQ, UK

**Keywords:** Progressive Palaeontology, Palaeontology, Palaeobiology, Conference report

## Abstract

The 20th instalment of the Progressive Palaeontology conference was held from 17th-20th of June 2024 at the University of Bristol, UK. Progressive Palaeontology gives postgraduate students experience of presenting at a conference to an audience of their peers, and the opportunity to form networks with researchers at their career stage. The conference was organised on behalf of the Palaeontological Association by Hady George, Thomas Farrell, James R. G. Rawson, Isaura Aguilar-Pedrayes, Benton Walters and Kirsten E. Flett, all of whom are postgraduate students in the Bristol Palaeobiology group. The meeting was a great success, featuring a high standard of research presentations on a wide range of topics, and inclusive and educational events hosted throughout the conference.

## Teaser

**Summary:** The 20th student-organised Progressive Palaeontology conference was held from 17th-20th of June 2024, giving postgraduate students experience of presenting at a conference to their peers. The organisers describe the meeting and offer advice for future meetings.

## Progressive Palaeontology 2024 conference report

Progressive Palaeontology (ProgPal) is a free annual conference for postgraduate and senior undergraduate students, organised by postgraduate students on behalf of The Palaeontological Association. The Palaeontological Association is a UK organisation that represents around 1000 worldwide members of all career stages, covering all aspects of the field of palaeontology. The Progressive Palaeontology conference provides an important venue for early career researchers to present their work to a community of their peers in a safe and inclusive environment. It is often the first opportunity that new researchers have to present their work outside their home institutions. ProgPal 2024 was the 20th instalment and was held at the University of Bristol, UK, from 17th-20th of June 2024. The conference was attended by 135 in-person and 7 virtual delegates, 129 of whom were from UK institutions. All the organisers were PhD students.

The presentations at ProgPal 2024 featured a diverse array of subjects ranging from pre-Cambrian trace fossils to the ecology of large predatory dinosaurs. Presentations consisted of posters, lightning talks (3 min for talk, 2 min for questions) and full talks (12 min for talks, 3 min for questions). The talks were organised into themed sessions: ‘Invertebrates and origins’, ‘Palaeoenvironment and climate’, ‘Anatomy, form, and function’, ‘Macroevolution and big data’, and ‘Palaeoecology’. An excellent keynote talk was given by Abi Crane (University of Southampton) entitled “What's in a Bird? Understudied Bird Anatomy Yields New Insights into Avian and Non-Avian Dinosaurs”. All presentations were of great quality, highlighting a bright future for palaeontology. Best presentation prizes were given to Campbell Marsden Hendrick (University of Bristol), for a poster on the ancient arthropod relative *Pambdelurion* Budd, 1998, and to Laura Cooper (University of Edinburgh) for a full talk on the enigmatic fossil fungus *Prototaxites* Dawson, 1859, from the Devonian of Scotland. Delegates who were not able to attend in person could engage with the conference through a dedicated server on the social media platform Discord.

**Fig. 1.**
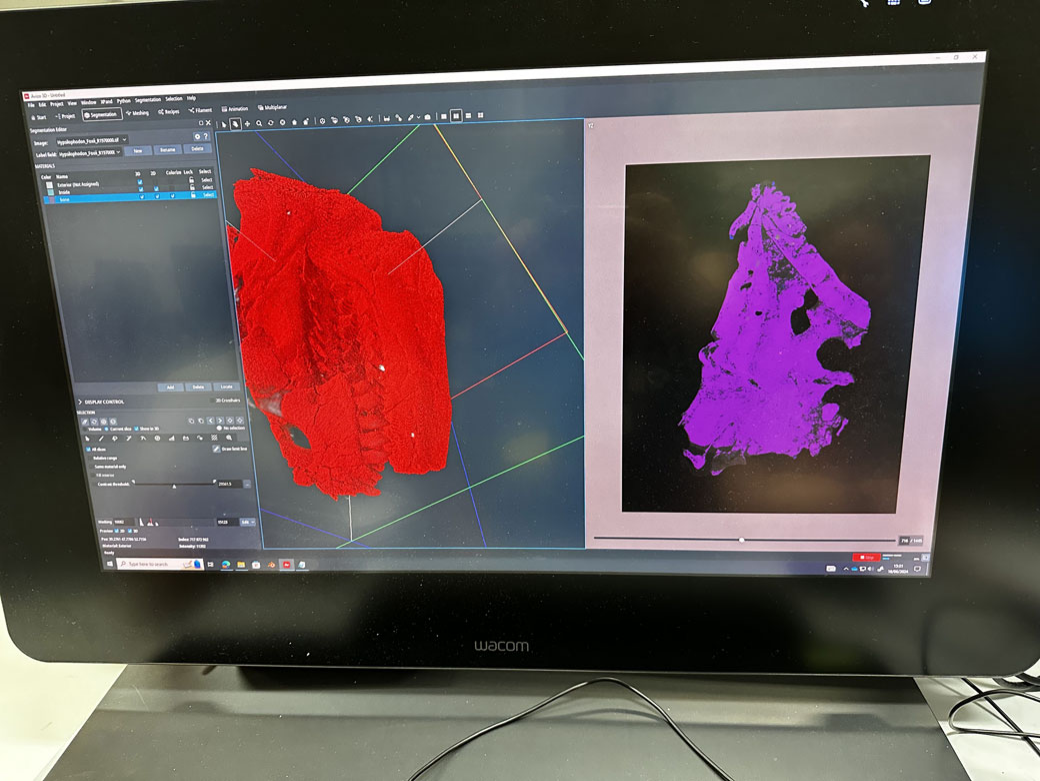
Segmentation of a specimen of the dinosaur *Thescelosaurus*, during the ProgPal 2024 Avizo workshop.

The first day of the conference was preceded by an optional evening of talks and discussion entitled “An insight into palaeomedia.” This event featured natural history filmmakers and consultants Dr Tom Fletcher, Zoe Cousins, Professor Emily Rayfield, Dr Tahlia Pollock, and Dr Andre Rowe. Delegates had a chance to listen to experts' experiences of working on natural history documentaries and receive advice on how to get involved themselves. This year's conference featured two workshops that delegates could attend depending on their research and career interests. One taught the basics of CT-data processing using Avizo (Thermo Fisher Scientific, 2021), and specifically focused on comparing the processing of CT-data of the head of the extant Komodo dragon and CT-data of the skull of the dinosaur *Thescelosaurus neglectus* Gilmore, 1913 (Fig. 1) to demonstrate the challenges of working with CT-data of fossils. The other workshop was a tour of the Earth Sciences collection of Bristol Museum & Art Gallery which provided delegates an insight into the management and curation of fossil and mineral museum collections.

**Fig. 2.**
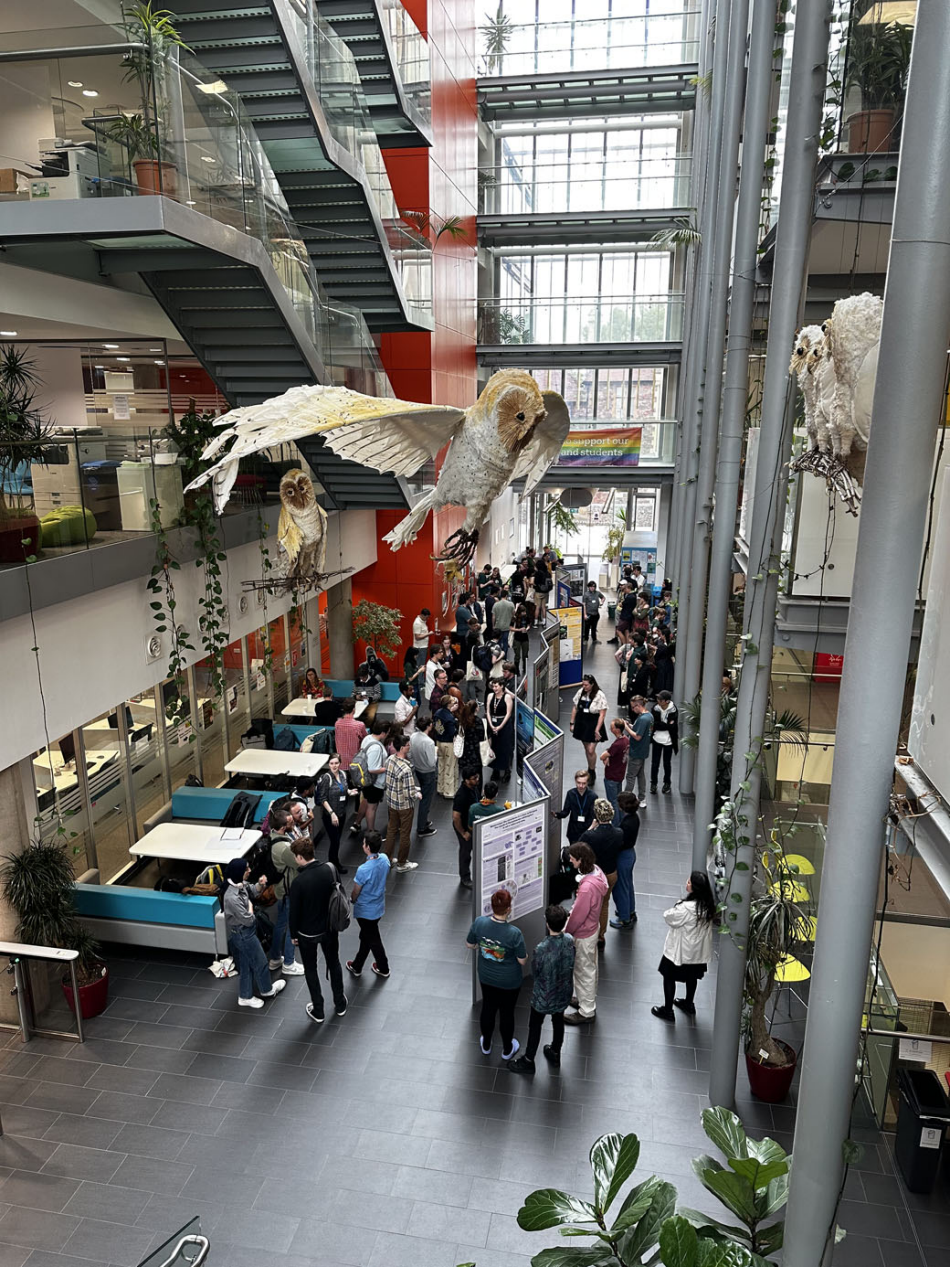
Poster session and icebreaker of ProgPal 2024 in the atrium of the Life Sciences Building of the University of Bristol.

The poster session on the first day of the conference gave the delegates the chance to practise presenting their work in a relaxed environment and encouraged them to connect with other researchers in their fields. An icebreaker event on the first day gave delegates a chance to meet each other before talks began (Fig. 2). The conference also included an LGBTQ+ meetup led by a University of Bristol postdoctoral researcher where attendees shared experiences in the field that have been shaped by their identity. The final talk session was followed by the giving of awards (mentioned above) and an auction of fossil-related art, books and memorabilia, which is a ProgPal tradition. Delegates could also attend the conference dinner at The Mall, Bristol, for additional networking opportunities.

The conference concluded with a field trip to fossiliferous units near Cardiff Bay in Penarth, Wales. Penarth is a very prolific collecting site for marine invertebrates such as bivalves, brachiopods, gastropods, ammonites and echinoids in Late Triassic - Early Jurassic coastal marine sediments. Delegates collected fossils such as invertebrate shells, fish teeth and fragments of marine reptiles, and visited a large, exposed dinosaur trackway on the beach thought to have been made by Triassic sauropodomorphs (Falkingham et al., 2022).

The high quality of presentations prepared delegates for presenting at larger conferences where senior experts will be present. The networking opportunities familiarised delegates with forming professional connections with researchers with similar research interests. Many attendees expressed that they had gained a combination of computational, curatorial, and/or fossil prospecting skills from the workshops and field trip of the conference that will be useful to them in their careers. Numerous attendees expressed that they deeply enjoyed the conference and were appreciative of its accessibility.

Much of the conference would not have been possible without the generous support of its sponsors. *PeerJ* awarded the two presentation prizes, whose previously mentioned winners received free publications in their journal. The Avizo workshop would not have been possible without Thermo Fisher Scientific providing trial licences for delegates and funding travel and accommodation for a representative to teach the workshop. The University of Bristol's Faculty of Science and Bristol Palaeobiology Research Group further assisted with funding and support. Funds were also supplied by the Palaeontological Association, which were used to provide conference souvenirs and refreshments for attendees. Generous funding from the Company of Biologists covered many travel grants for attendees as well as the coach required for the field trip, which greatly increased the accessibility of the conference.

Overall, ProgPal 2024 was highly successful thanks to the excellent presentations by attendees and the funding and support of several university and external organisations. Attendees provided a great amount of positive feedback, with some even enquiring into how to apply to host next year's iteration of the conference. As the committee, we have gained the invaluable experience of organising a scientific conference and the joys and difficulties associated with it. Our advice for future organisers includes:

(1) Become acquainted with your institution's room-booking system and book suitably sized rooms as soon as possible. This includes rooms for talks, poster sessions, satellite meetings and icebreaker/networking sessions. Use lecture theatre capacity as a limit on the number of attendees.
(2) Apply for funding from sources used by past meetings but also explore new sources. Contact publishers, learned societies and departments in your institution which may have money available even if they do not advertise it.
(3) Think about the unique mobility challenges of the city or campus in which your meeting is happening and make travel assistance available.
(4) Provide a decision on abstracts as soon as possible to allow delegates to book travel and accommodation early.

We believe the accessibility of our conference, its diverse range of events, and the acquisition of funding from a broad range of sources sets an example for future student conferences to follow.

## References

[BIO061822C1] Budd, G. E. (1998). Stem group arthropods from the Lower Cambrian Sirius Passet fauna of north Greenland. In *Arthropod Relationships*, pp. 125-138. Dordrecht: Springer Netherlands.

[BIO061822C2] Dawson, J. W. (1859). On fossil plants from the Devonian rocks of Canada. *Quart. J. Geol. Soc.* 15, 477-488. 10.1144/GSL.JGS.1859.015.01-02.57

[BIO061822C3] Falkingham, P. L., Maidment, S. C., Lallensack, J. N., Martin, J. E., Suan, G., Cherns, L., Howells, C. and Barrett, P. M. (2022). Late Triassic dinosaur tracks from Penarth, south Wales. *Geol. Mag.* 159, 821-832. 10.1017/S0016756821001308

[BIO061822C4] Gilmore, C. W. (1913). A new dinosaur from the Lance Formation of Wyoming: Smithsonian Miscellaneous Collection, v. 61 No. 5, pp.1-5.

